# Within‐generation consequences of postsettlement mortality for trait composition in wild populations: An experimental test

**DOI:** 10.1002/ece3.4911

**Published:** 2019-02-13

**Authors:** Benjamin J. Ciotti, Serge Planes

**Affiliations:** ^1^ Laboratoire d'excellence "CORAIL" USR 3278 CNRS‐EPHE‐UPVD CRIOBE Perpignan France; ^2^ School of Biological and Marine Sciences University of Plymouth Plymouth UK

**Keywords:** delayed life‐history effects, genetic sweepstakes, local adaptation, plasticity, recruitment, selection, trait‐mediated effects

## Abstract

There is a critical need to understand patterns and causes of intraspecific variation in physiological performance in order to predict the distribution and dynamics of wild populations under natural and human‐induced environmental change. However, the usual explanation for trait differences, local adaptation, fails to account for the small‐scale phenotypic and genetic divergence observed in fishes and other species with dispersive early life stages. We tested the hypothesis that local‐scale variation in the strength of selective mortality in early life mediates the trait composition in later life stages. Through in situ experiments, we manipulated exposure to predators in the coral reef damselfish *Dascyllus aruanus* and examined consequences for subsequent growth performance under common garden conditions. Groups of 20 recently settled *D. aruanus* were outplanted to experimental coral colonies in Moorea lagoon and either exposed to natural predation mortality (52% mortality in three days) or protected from predators with cages for three days. After postsettlement mortality, predator‐exposed groups were shorter than predator‐protected ones, while groups with lower survival were in better condition, suggesting that predators removed the longer, thinner individuals. Growth of both treatment groups was subsequently compared under common conditions. We did not detect consequences of predator exposure for subsequent growth performance: Growth over the following 37 days was not affected by the prior predator treatment or survival. Genotyping at 10 microsatellite loci did indicate, however, that predator exposure significantly influenced the genetic composition of groups. We conclude that postsettlement mortality did not have carryover effects on the subsequent growth performance of cohorts in this instance, despite evidence for directional selection during the initial mortality phase.

## 1 INTRODUCTION

While physiological traits have long been known to vary among members of the same species, systematic spatial variations in the performance of wild populations have recently become a major research focus (Gaston et al., 2009). Local adaptation, for example, can drive important spatial differences in phenotypes (Conover, 1992; Conover, Brown, & Ehtisham, 1997), controlling not only how populations and communities respond to environmental change (Beckerman, Benton, Ranta, Kaitala, & Lundberg, 2002; Calosi et al., 2017; Giménez, 2004; Pechenik, Wendt, & Jarrett, 1998) but also their functional role within ecosystems (Bassar et al., 2010). Patterns and causes of these “intrinsic” sources of variation in physiological performance are critical for understanding the dynamics of populations, communities, and ecosystems, and for predicting responses to future environmental scenarios.

The processes that cause populations to diverge at ecological scales have received relatively little attention in ecophysiological studies, and compelling evidence now suggests that local adaptation is not always a sufficient explanation. Local adaptation occurs when barriers to dispersal are sufficient to allow phenotypic differences to accumulate through natural selection (Kawecki & Ebert, 2004). However, phenotypic divergence is being observed at small spatial scales and within well‐mixed populations where such barriers are lacking (Richardson, Urban, Bolnick, & Skelly, 2014). Until recently, for example, the prevailing view was that spatial divergence in high fecundity species, such as many marine fishes and invertebrates, would be homogenized by dispersive early life stages. Overwhelming evidence now supports intraspecific divergence in these groups at surprisingly small scales (Conover, Clarke, Munch, & Wagner, 2006; Sanford & Kelly, 2011; Wennerström et al., 2013). We need other explanations, beyond local adaptation, to explain such instances of phenotypic and genotypic divergence.

Mechanisms to produce phenotypic divergence against a backdrop of high gene flow have been suggested but have received relatively little attention to date. One suggestion is that the phenotypic and genetic composition of populations reflects not just adaptation across generations, but also the signature of within‐generation selective bottlenecks experienced during early life (Sanford & Kelly, 2011; Sotka, 2012). These phenotypic differences can persist as “balanced polymorphisms” that are renewed each generation without necessarily leading to longer‐term evolutionary change (Bourret, Dionne, & Bernatchez, 2014; Laporte et al., 2016; Sanford & Kelly, 2011). In other words, strong selection on a cohort during early life stages could shape later performance, even when populations are completely mixed each generation. Such mechanisms could have important ecological implications at small scales (Flowers, Schroeter, & Burton, 2002; Lande & Arnold, 1983; Siepielski, DiBattista, & Carlson, 2009) and would prevail in taxa with strong, selective, early‐life mortality (Sotka, 2012).

Species with high fecundity are particularly predisposed to strong, selective, early‐life mortality. Massive overproduction of offspring leads to narrow, selective bottlenecks in early life (Houde, 1989; Roughgarden, Gaines, & Possingham, 1988; Sogard, 1997). For example, larval and juvenile fishes experience strong selection on size (Carr & Hixon, 1995; Holmes & McCormick, 2006; McCormick & Meekan, 2007), nutritional condition (Booth & Beretta, 2004; Hoey & McCormick, 2004), and growth rate (Houde, 1997; Searcy & Sponaugle, 2001; Sogard, 1997; Takasuka, Aoki, & Oozeki, 2007), particularly at the larval–juvenile transition when individuals settle to benthic habitats (Doherty et al., 2004; Hoey & McCormick, 2004; Schmitt & Holbrook1999b, ; Steele & Forrester, 2002). Indeed, this process of selective mortality is so integral to our understanding of high fecundity species that it forms the basis for theories of population regulation (Houde, 1989). Early‐life mortality therefore has potential to exert strong leverage over the phenotypic composition of adult stages such that even small differences in the strength and direction of selection could produce microgeographic variation in phenotypes.

Processes of selective mortality are particularly well documented in young reef fishes. Field experiments and longitudinal analyses of otolith microstructure have demonstrated that survival is often enhanced by large size (McCormick & Hoey, 2004; Schmitt & Holbrook1999b, b; Vigliola & Meekan, 2002) or high nutritional condition (Booth & Beretta, 2004; Figueira, Booth, & Gregson, 2008). Fish that are larger or in better nutritional condition may be better competitors for predator‐free shelter space (Booth & Beretta, 2004; Holbrook & Schmitt, 2002), take fewer risks to obtain food (Booth & Beretta, 2004), and escape predators more effectively (Sogard, 1997). Other studies, however, have found that survival is favored by small size and low nutritional condition (Gagliano, McCormick, & Meekan, 2007; Litvak & Leggett, 1992; Sogard, 1997). In fact, even within the same species, the target, strength, and direction of selection can be variable in time and space depending on the initial phenotypic composition (Sponaugle & Grorud‐Colvert, 2006), abiotic conditions (Holmes & McCormick, 2006), and interactions with conspecifics (McCormick & Meekan, 2007) or other species (Carr & Hixon, 1995; Figueira et al., 2008; Takasuka et al., 2007). The variable nature of selective mortality at settlement in reef fish now provides a useful framework to explore the carryover consequences for performance in later life stages.

Despite the evidence that selective mortality influences standing genetic and phenotypic variation in early life, little is known about consequences for subsequent physiological performance. Selection on size, condition, and prior growth rate has been documented, but whether this leads to long‐term shifts in the subsequent growth rate of cohorts has rarely been tested directly (Fox et al., 2014; McCormick & Hoey, 2004; Vigliola & Meekan, 2002). The existence and nature of these carryover effects will depend on the plasticity in the trait under selection. For example, the extent to which selection on size modifies the growth potential of a settling cohort depends on whether size variation results from persistent, intrinsic (e.g., heritable traits) or reversible, extrinsic (e.g., environmental experience) differences in growth performance.

**Figure 1:**
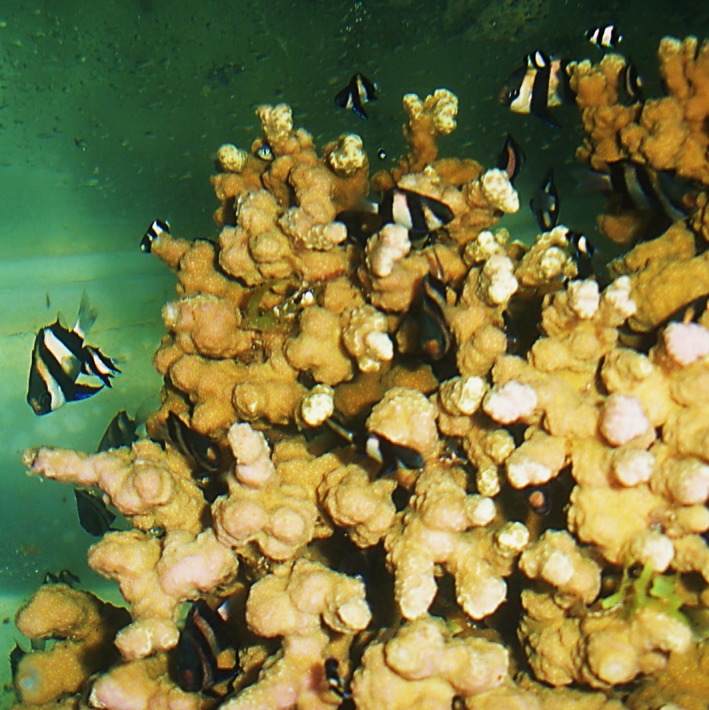
*Dascyllus aruanus* on *Porites rus*at Moorea, French Polynesia. Photograph: B. Ciotti

We conducted manipulative experiments with the coral reef damselfish *Dascyllus aruanus*(Figure 1) to explore the role of postsettlement mortality as a “gatekeeper” to the genotypes and phenotypes present at later life stages. Specifically, we tested the hypothesis that postsettlement mortality influences growth rate during the subsequent juvenile stage. We manipulated *D. aruanus*cohorts on natural corals, either protecting or exposing them to predators immediately after settlement. Predator exposure influenced the average size of surviving cohort members, either due to selection for size or due to plasticity in growth among treatments. Although we could not manipulate or measure selection directly, postsettlement mortality is known to impose directional selection in *Dascyllus* spp. (Holbrook & Schmitt, 2002; Schmitt & Holbrook1999b, b) and we consider this the most likely explanation for the size differences we observed. We then examined how this new distribution of phenotypes influenced subsequent growth by comparing common garden growth performance and genetic composition of the survivors. Our study provides one of the first direct tests of the consequences of early‐life mortality for subsequent physiological performance in wild populations of high fecundity species.

## 2 MATERIALS AND METHODS

### 2.1 Experimental design

We tested how postsettlement mortality influenced subsequent growth of *D. aruanus*at Moorea, French Polynesia (17°30′S, 149°50′W). *D. aruanus* has been used extensively as a model to understand settlement and recruitment processes (Forrester, 1990; Jones, 1997; Pini, Planes, Rochel, Lecchini, & Fauvelot, 2011; Schmitt & Holbrook1999b, b). It hatches from benthic eggs and has a *ca.* three‐week planktonic larval stage (Brothers, Williams, & Sale, 1983; Wellington & Victor, 1989). Settlement occurs in discrete, semilunar pulses (Schmitt & Holbrook1999c, c), after which individuals show high site‐fidelity to individual coral colonies (Forrester, 1990; Sale, 1971; Schmitt & Holbrook1999b, b). Previous experiments with *Dascyllus*spp. at Moorea demonstrate high postsettlement mortality due to predation by fishes at dusk (Holbrook & Schmitt, 2002, 2003; Schmitt & Holbrook1999b, b). Mortality in the wild varies with predator density and can be manipulated experimentally with predator exclusion cages (Holbrook & Schmitt, 2003). The biology of *D. aruanus* therefore allows discrete groups of settlers to be experimentally exposed to short periods of mortality and consequences for growth to be monitored throughout time, under natural conditions. We worked in accordance with national regulations for vertebrate manipulation (qualification number 00675 from the Ministry of Agriculture, awarded to Dr. Serge Planes).

**Figure 2:**
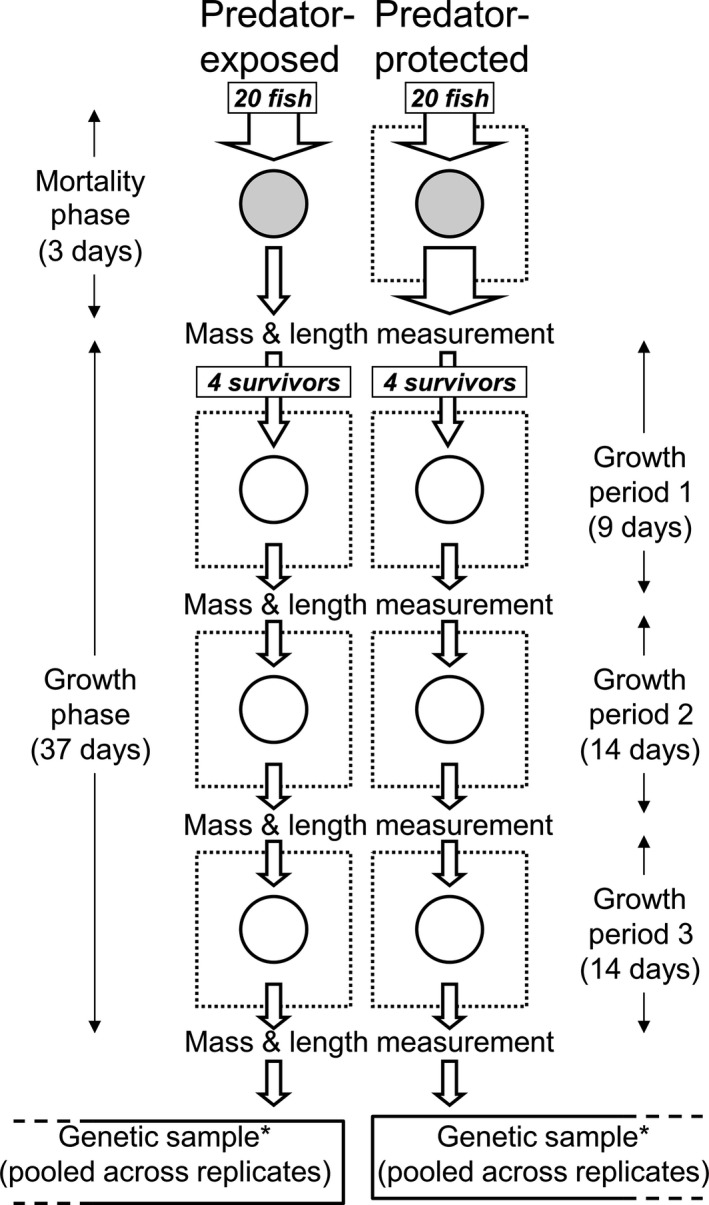
Experimental procedure for one replicate group from both predation treatments. During an initial “mortality phase,” 20 recently settled *Dascyllus aruanus*, outplanted to *Pocillopora eydouxi* colonies (gray circle), were either covered with a predator exclusion cage (dotted square, predator‐protected treatment) or left open (predator‐exposed treatment). During the subsequent “growth phase,” a subset of four survivors from each group was grown on *Porites rus* colonies (open circle) in predator exclusion cages for three consecutive growth periods. Individual body mass and standard length were measured after the mortality phase and after each growth period to test whether mortality was selective and explore consequences for subsequent growth. Genetic composition was compared between survivors of each treatment. The entire procedure was run in parallel for five replicate groups per treatment. *Sample for genetic analysis taken either at the end of the mortality phase or growth phase

Our experiment involved two distinct, but consecutive phases (Figure 2). In the first, the “mortality phase,” replicate groups of *D. aruanus*experienced one of two predation treatments, created using predator exclusion cages (*n* = 5 groups per treatment, Figure 2). In the second, the “growth phase,” growth and nutritional condition of surviving members of these groups were measured under common garden conditions for three consecutive growth periods (Figure 2). Therefore, treatments differed only by the presence or absence of a predator exclusion cage during the short, initial mortality phase.

### 2.2 Mortality phase

On 23 June 2009, and 24 June 2009, 251 newly settled *D. aruanus* were collected from corals using clove oil and nets. Regular monitoring of reef sites suggested that settlement had occurred less than 48 hr prior to collection. Fish were briefly held in aquaria supplied with ambient lagoon water until outplanting.

On June 26, groups of 20 randomly selected *D. aruanus* (mean ± *SD* standard length = 8.53 ± 0.450 mm) were outplanted onto each of 10 *Pocillopora eydouxi*colonies (*ca.* 30 cm diameter, *ca.*20 cm high) in an area of patch reef (2–3 m depth) in the lagoon. The density of outplants approaches the upper end of the range of natural settlement densities at Moorea (personal observation; Schmitt & Holbrook1999a, a). Host *P. eydouxi*colonies sat on cinder blocks on bare sand at least 5 m from other reef structures, to discourage *D. aruanus* from emigrating. Each colony had been emptied of resident fish and supplied with one adult *D. aruanus* to improve apparent habitat suitability. Monitoring of two additional groups of *D. aruanus* that were submerged at the release site and then returned to the laboratory suggested that mortality due to experimental manipulation was minimal: Fish displayed normal behavior and high survival (95%) over the following five days.

Outplants were acclimatized during the first night after release by enclosing host colonies in cages (2 mm mesh, 0.5 × 0.5 × 0.5 m dimensions). The following morning colonies were randomly assigned to one of two predation treatments for a three‐day mortality phase: Acclimatization cages were either removed (“predator‐exposed” treatment) or replaced with a predator exclusion cage (“predator‐protected” treatment; cage = 6 mm wire mesh, 0.5 × 0.5 × 0.5 m dimensions). *D. aruanus* could pass through the mesh of predator exclusion cages, but rarely did so (personal observation). Experimental colonies were visited daily to count survivors and clean cages. On 30 June 2009, three days after initiation of the predation treatments, surviving *D. aruanus* were removed, counted, and returned to the laboratory where they were weighed and photographed (for length measurement). The growth phase was initiated on the following morning.

### 2.3 Growth phase

In order to test our hypothesis that post‐settlement predation mortality influences growth performance of the surviving cohort, common garden growth rates were compared between predator‐exposed and predator‐protected groups following the mortality phase. Densities were standardized by randomly selecting four survivors from each of the 10 groups for the growth phase (40 fish in total, *n* = 5 groups per treatment). Growth rates of each group were then measured for three consecutive periods lasting nine (growth period one) or 14 (growth periods two and three) days.

Groups were grown on separate *Porites rus*colonies in a sandy area of the lagoon (2–3 m depth). *P. rus*is a robust coral that is effective at sheltering *D. aruanus* from predators, so was considered suitable for the extended growth phase. Colonies had been emptied of resident fish and supplied with an adult *D. aruanus*. Groups of *D. aruanus*were randomly assigned to one of the *P. rus* colonies (*ca.* 30 cm diameter, *ca.*25 cm high), which were placed directly on the sand in a 2 × 5 grid with 8 m spacing. All groups were protected by predator exclusion cages throughout the growth phase and were provided with acclimatization cages on the first night after outplanting. Colonies were visited daily to clean cages, count *D. aruanus* and remove any new settlers. At the end of each growth period, experimental fish were weighed and photographed (for length measurement) in the laboratory then returned to host colonies on the same day. Mortality during the growth phase was minimal: Two of the 40 outplanted fish died or disappeared during the first growth period, three during the second, and seven during the third. These 12 losses were equally divided between predation treatments.

Size and nutritional condition of *D. aruanus* at the end of the mortality and growth phases were compared between predation treatments and with respect to the number of survivors. Mass (g) and standard length (mm, from photographs) were measured and used to calculate morphometric condition factor (=10^5^ × mass × standard length^‐3.08^; hereafter “condition”). The scaling exponent for condition was obtained from standard major axis regression of ln(mass) against ln(standard length) of the experimental fish. Statistical analyses were performed using mean measurements for each group (*n* = 5 groups per treatment) since groups were the scale at which predation treatments were replicated. Variance in mass of young fish tends to be generated by differences in exponential growth rates, so mass was log‐transformed (“ln(mass)”) for statistical tests. Survival was calculated as the number of individuals counted upon retrieval of groups at the end of the mortality phase. Compliance with assumptions of normality and homoscedasticity was assessed from residual plots. We then used *t* tests (Welch's *t* tests where variances appeared heterogeneous) to test how ln(mass), standard length, and condition differed between the two predation treatments. We used Pearson's product moment coefficients to examine correlations with survival.

We examined whether changes in ln(mass), standard length, and condition of groups during the growth phase were related to the history of predator exposure and mortality during the preceding mortality phase. Daily increments in ln(mass) (= “instantaneous growth rate,” d^‐1^), standard length (= “linear growth rate,” mm), and condition were calculated from group means for each period of the growth phase (*n* = 5 groups). Differences in these metrics among predation treatments and growth periods were tested using groups by trials repeated‐measures ANOVA (Quinn & Keough, 2002) with group as the random “subjects” factor, growth period as the fixed “within‐subjects” factor, and predation treatment as the fixed “between‐subjects” factor. Compliance with assumptions of normality and homoscedasticity was assessed from residual plots.

### 2.4 Genetic analysis

To establish how mortality influenced the genetic composition of groups, 116 of the 121 survivors from the mortality phase (five samples that were outplanted during the growth phase were not recovered) were screened at 10 microsatellite loci. This included predator‐protected groups, representing the initial diversity, as well as the survivors of predator exposure. Caudal fin and posterior muscle tissue were digested in 420 μl Qiagen DX Tissue Digest (Qiagen, Courtabœuf, France) at 55°C for three hours, and DNA was extracted using a QIAxtractor with CorProtocol 14202 (Qiagen). Microsatellite loci (Da304, Da331, Da360, Da494, Da523, Da542, Da565, Da589, Da590, Da593) were amplified using a Multiplex PCR Kit (Qiagen) with primer combinations, dyes, and annealing temperatures as described by Fauvelot, Smith‐Keune, Jerry, Buston, and Planes (2009). Amplified fragments were separated against a 400‐bp internal size standard and scored in a Beckman Coulter CEQ™ 8000 Genetic Analysis System (Beckman Coulter, Roissy, France).

We checked for scoring errors due to stuttering, large allele dropout, and null alleles using MICRO‐CHECKER Version 2.2.3 (van Oosterhout, Hutchinson, Wills, & Shipley, 2004). Gametic disequilibrium and deviations from Hardy–Weinberg expectations were tested with exact tests based on Markov Chain procedures in GENEPOP Version 4.0.10 (Raymond & Rousset, 1995) with sequential Bonferroni correction (Rice, 1989) to ensure that treatment‐wide *α* = 0.05.

Following tests for procedural errors and genetic disequilibrium, we compared the genetic composition of the two treatments. Allelic richness was calculated by rarefaction in Fstat Version 2.9.3.2 (Goudet, 1995). The number of alleles, observed and expected heterozygote frequencies, tests of population differentiation (F_ST_), and relatedness were calculated in GENALEX Version 6.501 (Peakall & Smouse, 2012). F_ST_ was calculated by AMOVA, and significance was tested against 9,999 random permutations. Sequential Bonferroni correction was applied to F_ST_ by locus to ensure that treatment‐wide *α* = 0.05 (Rice, 1989). Relatedness and associated 95% confidence intervals (10,000 bootstraps) were calculated from the mean of the Queller and Goodnight (1989) pairwise estimator for each treatment.

## 3 RESULTS

### 3.1 Mortality phase

**Figure 3:**
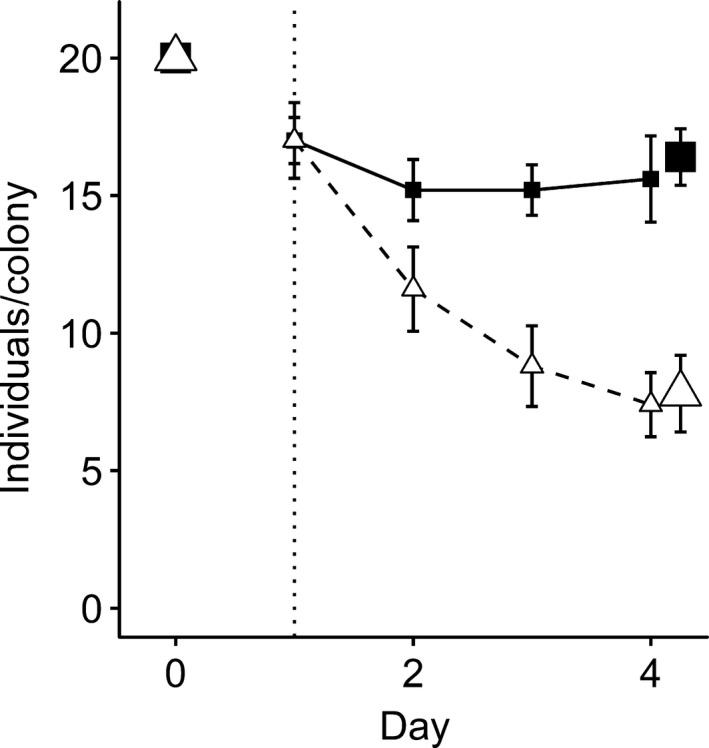
Mortality of recently settled *Dascyllus aruanus* during predation treatments. Ten groups of 20 *D. aruanus* were outplanted to individual *Pocillopora eydouxi*colonies on 26 June 2009 (day 0), acclimatized for one day and then assigned to a predator‐protected (solid line, black squares, *n* = 5) or predator‐exposed (dashed line, white triangles, *n* = 5) treatment for three days. Lines and small symbols represent in situ daily visual counts (mean ± *SE*). Large points represent direct counts (mean ± *SE*) after removing groups from host colonies: The final date is slightly offset, for visual clarity. Dotted vertical line indicates the end of the acclimatization period and the initiation of predation treatments

The three‐day mortality phase produced large differences in survival between predation treatments. Approximately 15% of fish disappeared during the initial overnight acclimatization, but losses were identical between treatments such that both contained a mean of 17.0 (± *SD *= 2.40) fish at the start of the experiment (Figure 3). Thereafter, counts of predator‐protected groups remained stable (mean ± *SD* at the end of the mortality phase = 16.4 ± 2.30; Figure 3). Meanwhile, predator‐exposed groups lost individuals daily and had half as many fish as the predator‐protected groups after three days (mean ± *SD *= 7.80 ± 3.11; Welch's *t* test,* t = *4.97,* df *= 7.37, *p* = 0.0014; Figure 3). Therefore, assuming that cages effectively eliminated predation and controlled for losses due to handling mortality and migration, predators removed 52.4% of individuals from predator‐exposed groups during the three‐day mortality phase.

**Figure 4:**
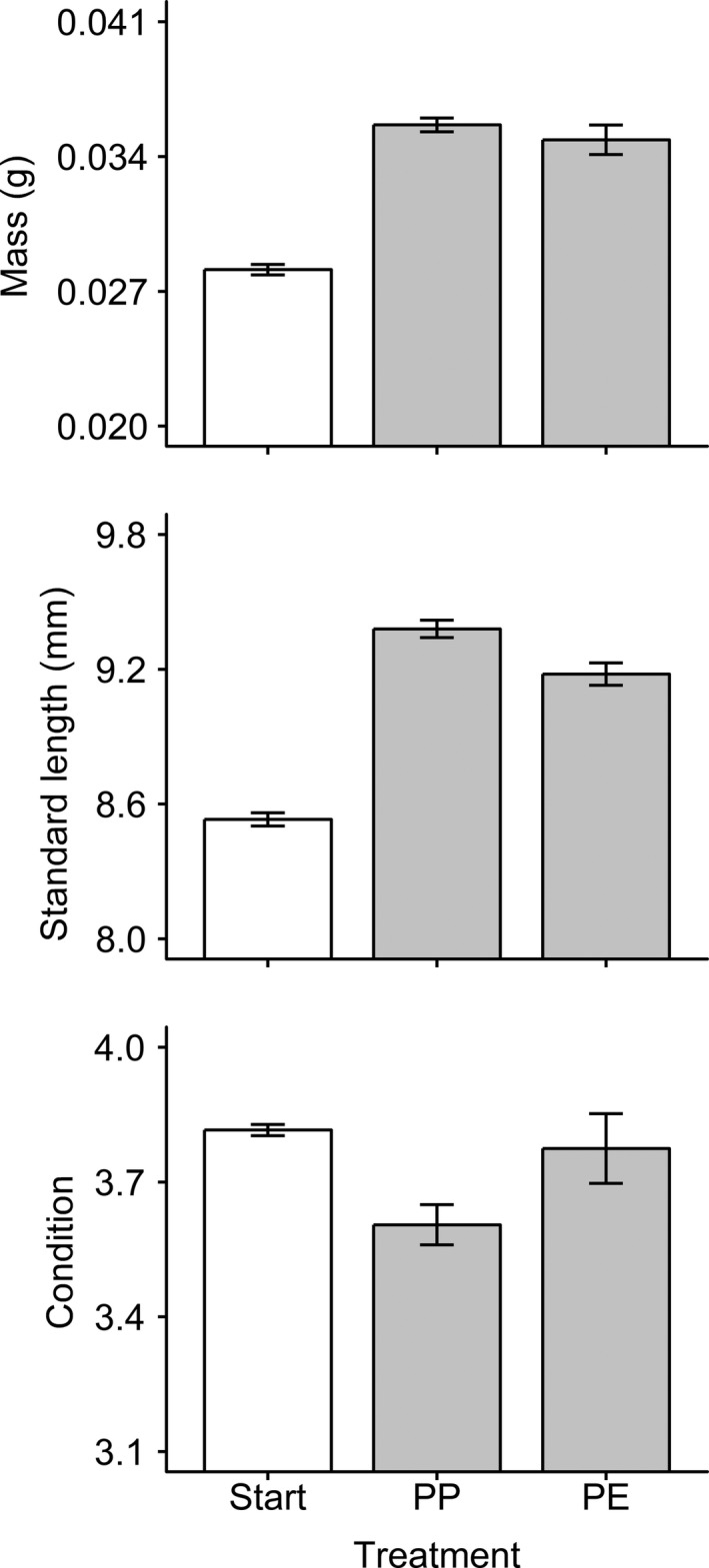
Size and nutritional condition of recently settled *Dascyllus aruanus* before and after predation treatments. Gray bars represent mean of replicate groups (± *SE*, *n* = 5) at the end of the mortality phase, by treatment (PE: predator‐exposed; PP: predator‐protected). White bar (mean ± *SE*, *n* = 2) represents initial size and morphometric condition factor at the start of the mortality phase

The standard length of *D. aruanus* at the end of the mortality phase differed between predation treatments. Predator‐exposed groups were 0.201 mm (standard length) shorter than predator‐protected groups (Welch's *t* test,* t = *3.19,* df *= 7.56, *p* = 0.014; Figure 4). No differences between predation treatments were detected for ln(mass) (Welch's *t* test,* t = *1.13, *df *= 4.94, *p* = 0.31) or condition (Welch's *t* test,* t = *1.89,* df *= 6.40, *p* = 0.10; Figure 4).

**Figure 5:**
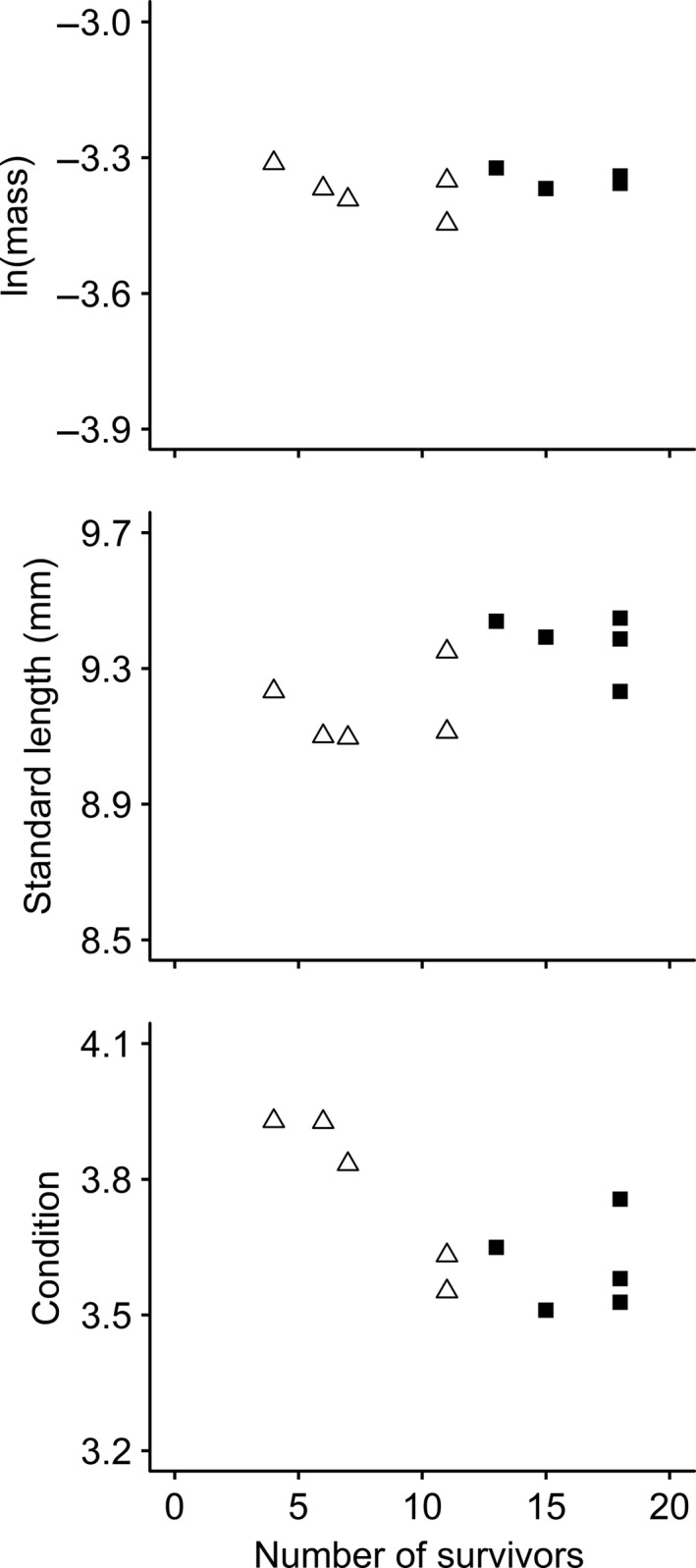
Size and nutritional condition against number of survivors in recently settled *Dascyllus aruanus*following predation treatments. Points represent group means. Treatments include predator exposure (white triangles) or predator protection (black squares)

Groups with lower survival were generally shorter and in better condition at the end of the mortality phase. There was a positive correlation between standard length and survival (*r* = 0.651,* t = *2.43,* df *= 8.00, *p* = 0.041), but this was largely driven by an overall treatment effect: The correlation was not statistically significant within the predator‐exposed (*r = *0.245,* t = *0.438,* df *= 3.00, *p* = 0.69) or the predator‐protected (*r *= −0.416,* t = *0.793,* df *= 3.00, *p* = 0.49) treatments alone (Figure 5). There was also a negative correlation between condition and survival across both treatments (*r *= −0.751,* t = *3.21,* df *= 8.00, *p* = 0.012; Figure 5). This was largely driven by a statistically significant correlation in the predator‐exposed treatment (*r *= −0.961,* t = *6.00,* df *= 3.00, *p* = 0.0093), but not the predator‐protected treatment (*r = *0.0650,* t = *0.113,* df *= 3.00, *p* = 0.92; Figure 5). There was no correlation between ln(mass) and number of survivors overall (*r = *0.0697,* t = *0.198,* df *= 8.00, *p* = 0.85), within the predator‐exposed treatment (*r *= −0.623,* t = *1.38,* df *= 3.00, *p* = 0.26) or within the predator‐protected treatment (*r *= −0.385,* t = *0.722,* df *= 3.00, *p* = 0.52; Figure 5).

### 3.2 Growth phase

**Figure 6:**
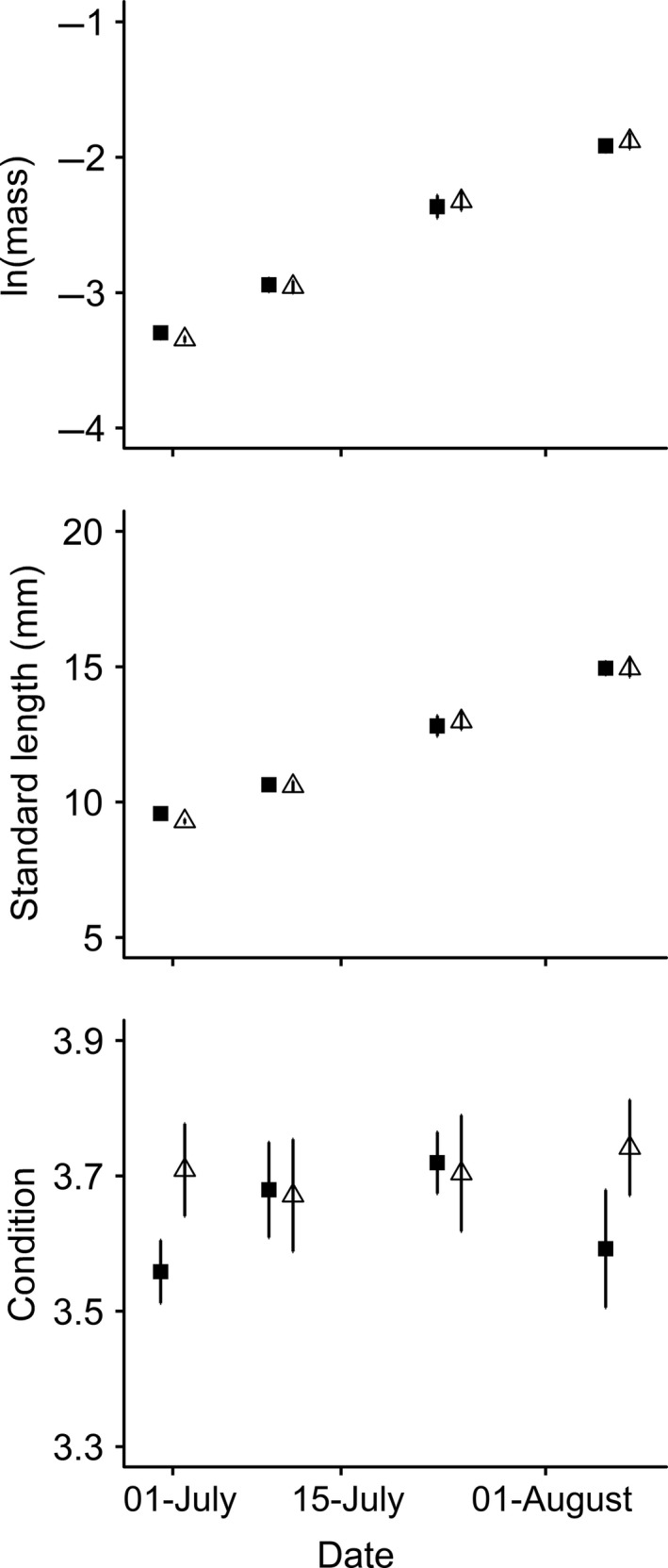
Effect of post‐settlement mortality on subsequent growth in groups of recently settled *Dascyllus aruanus*. Points represent means of replicate groups (± *SE*, *n* = 5) that were predator‐exposed (white triangles) or predator‐protected (black squares) during a prior three‐day mortality phase. Error bars are small and do not extend beyond mean symbols in some cases. Dates are slightly offset for visual clarity

Fish originating from both predation treatments grew rapidly and at similar rates across each of the three growth periods (Figure 6). Mean ± *SD* standard length increased from 9.43 ± 0.658 mm to 14.9 ± 1.34 mm during the growth phase. Mass increased from 0.0367 ± 0.00700 g to 0.152 ± 0.0363 g. Groups by trials repeated‐measures ANOVA suggested similarity across growth periods in linear growth rate and condition changes (Figure 6; Table 1). Instantaneous growth rate was, however, lower in the final period compared to the preceding two (Figure 6; Table 1; Tukey's HSD, *p* < 0.023). No significant treatment effects or interactions between treatment and growth period were detected for any of the metrics of growth examined (Figure 6; Table 1). Therefore, short‐ and medium‐term growth performances of groups that had previously been exposed to predation mortality were similar to predator‐protected controls.

**Table 1:** Groups by trials repeated‐measures ANOVA of size and nutritional condition changes in recently settled* Dascyllus aruanus*following predation treatments

Response	Factor	MS (×10^4^)	*df*	*F*	*p*
∆ ln(Mass)	Treatment	0.459	1	1.13	0.32
	Group (Treatment)	0.404	8	–	–
	Period	3.70	2	5.35	0.017
	Treatment x Period	0.145	2	0.210	0.81
	Group (Treatment) x Period	0.691	16	–	–
∆ Standard Length	Treatment	7.11	1	0.763	0.41
	Group(Treatment)	9.31	8	–	–
	Period	25.1	2	1.74	0.21
	Treatment x Period	9.65	2	0.670	0.53
	Group (Treatment) x Period	14.4	16	–	–
∆ Condition	Treatment	0.340	1	0.441	0.53
	Group (Treatment)	0.771	8	–	–
	Period	1.64	2	0.409	0.67
	Treatment x Period	5.45	2	1.36	0.28
	Group (Treatment) x Period	4.01	16	–	–

Note

Changes in mean size and condition for 10 groups (“Group”; random “subjects” factor) were measured over three consecutive 9‐ to 14‐day growth periods (“Period”; fixed “within‐subjects” factor) following exposure to one of two predation treatments (“Treatment”; fixed “between‐subjects” factor). Predation treatments consisted of predator exposure or predator protection during an earlier three‐day mortality phase.

Similarity in growth performance between fish originating from the two predation treatments was corroborated by similarity in final size and condition at the end of the 37‐day growth phase. No differences in ln(mass) (*t* test,* t = *0.492,* df *= 8.00, *p* = 0.64), standard length (*t* test,* t = *0.00920,* df *= 8.00, *p* = 0.99), or condition (*t* test,* t = *1.33,* df *= 8.00, *p* = 0.22) were detected between predator‐exposed and predator‐protected groups (Figure 6). Furthermore, survival during the mortality phase did not correlate with ln(mass) (*r = *0.111,* t = *0.316,* df *= 8.00, *p* = 0.76), standard length (*r = *0.277,* t = *0.814,* df *= 8.00, *p* = 0.44), or condition (*r *= −0.504,* t = *1.65,* df *= 8.00, *p* = 0.14) at the end of the growth phase. Therefore, post‐settlement mortality did not have consequences for average size and condition of *D. aruanus* 37 days later.

### 3.3 Genetic analysis

No scoring errors or instances of large allele dropout were revealed with MICRO‐CHECKER for any locus in either treatment. A heterozygote deficiency was detected at Da494 for the predator‐exposed treatment, but no other heterozygote deficiencies were present (Table 2). Four pairs of loci were at gametic disequilibrium (exact test, *α* = 0.05 with sequential Bonferroni correction). These disequilibria involved either locus Da494 (in two of the pairs) or Da542 (in three of the pairs). Therefore, microsatellite data generally met assumptions for subsequent statistical tests, although there is some evidence of nonindependence between loci involving either Da494 or Da542.

**Table 2:** Genetic diversity of recently settled *Dascyllus aruanus* at 10 microsatellite loci following predation treatments

Locus	Treatment	*N*_a_	AR	*H*_o_	*H*_e_	*F*_is_	*F*_st_
Da565	PP	13	10.47	0.78	0.79	0.022	−0.005
	PE	9	9.00	0.75	0.78	0.055	
	All	14	10.08	0.77	0.79	0.030	
Da360	PP	10	8.75	0.86	0.80	−0.069	0.000
	PE	10	10.00	0.83	0.82	−0.006	
	All	12	9.59	0.85	0.81	−0.049	
Da494	PP	26	19.35	0.93	0.89	−0.035	0.025*
	PE	19	19.00	0.67	0.79	0.171*	
	All	27	19.18	0.84	0.87	0.036	
Da589	PP	23	19.65	0.96	0.94	−0.024	−0.003
	PE	17	17.00	0.94	0.92	−0.015	
	All	24	18.93	0.96	0.93	−0.022	
Da331	PP	6	4.90	0.74	0.69	−0.064	0.010
	PE	4	4.00	0.75	0.68	−0.095	
	All	6	4.62	0.74	0.69	−0.069	
Da304	PP	11	8.73	0.81	0.79	−0.028	0.014
	PE	9	9.00	0.78	0.81	0.053	
	All	12	8.64	0.80	0.80	0.004	
Da590	PP	25	20.00	0.99	0.92	−0.067	0.002
	PE	21	21.00	0.89	0.91	0.039	
	All	28	20.43	0.96	0.92	−0.034	
Da593	PP	21	16.99	0.90	0.87	−0.027	−0.004
	PE	17	17.00	0.78	0.87	0.119	
	All	23	17.08	0.86	0.87	0.017	
Da542	PP	31	23.30	0.90	0.93	0.043	−0.004
	PE	22	22.00	0.97	0.93	−0.031	
	All	32	22.55	0.92	0.94	0.018	
Da523	PP	18	14.21	0.81	0.85	0.045	0.016
	PE	13	13.00	0.81	0.76	−0.050	
	All	20	13.77	0.81	0.83	0.025	

Note

AR: allelic richness;* F*_is_: fixation index;* F*_st_: population differentiation by allele; *H*_e_: expected heterozygosity;* H*_o_: observed heterozygosity; *N*_a_: number of alleles; PE: predator‐exposed treatment (*n* = 36); PP: predator‐protected treatment (*n* = 80). All = both treatments combined (*n* = 116).

* Significant *F* values (*α* = 0.05) after sequential Bonferroni correction for multiple tests across loci, within treatments.

Allelic richness and observed heterozygosity differed slightly between predation treatments. Mean allelic richness over all loci was lower in the predator‐exposed treatment (14.1) relative to predator‐protected controls (14.6), although these differences were not statistically significant (paired *t* test,* t = *1.40,* df *= 9.00, *p* = 0.097). Four specific loci showed the reverse trend (Table 2). Observed heterozygosity of predator‐exposed fish was lower than predator‐protected fish at eight of the 10 loci, the largest reductions being 28% and 14% at Da494 and Da593, respectively (Table 2). The reduction in heterozygosity at Da494 resulted in a significant departure from Hardy–Weinberg equilibrium (Table 2).

Variance between treatments constituted a small (0.5%) but significant proportion of total genetic variance across all loci (AMOVA, *F*_ST _= 0.0049, *p* = 0.014). Analysis by locus indicated that these genetic differences were driven by Da494 (Table 2). Rerunning analyses on datasets balanced by random removal of samples from the predator‐protected treatment confirmed that genetic differentiation was not an artifact of uneven sample numbers between treatments. Mean pairwise relatedness was higher in predator‐exposed fish (0.0082) than predator‐protected fish (−0.011). Bootstrapped 95% confidence intervals around these means did not overlap, demonstrating that this difference was statistically significant. In summary, predators removed related genotypes nonrandomly and changed the genetic composition of groups.

## 4 DISCUSSION

This study investigated the role of post‐settlement mortality as a mechanism generating small‐scale physiological and genetic divergence in high fecundity populations. Specifically, we tested whether predator‐induced mortality affected growth performance during the subsequent juvenile stage. Through cage manipulations, we found that groups exposed to predators for three days were shorter, in better condition and genetically distinct from predator‐protected controls. These phenotypic and genetic differences are consistent with the removal of thinner individuals by predators and directional selection on phenotypic traits linked to the microsatellite markers. Despite this evidence that post‐settlement mortality imposed directional selection, common garden growth rates of *D. aruanus* groups were not influenced by prior mortality experience. In this instance, predator‐induced mortality did not carry over to shape the physiological performance of juveniles.

We attribute the marked losses of *D. aruanus* from predator‐exposed groups during the mortality phase of our experiment to predation mortality. Even though *D. aruanus*could move outside predator exclusion cages, losses from control colonies were minimal after acclimatization, consistent with previous observations that *D. aruanus* rarely migrate to adjacent structures (Forrester, 1990; Jones, 1987; Sale, 1971; Schmitt & Holbrook, 1999b). Predator‐exposed groups lost fish every day, suggesting sustained predation rather than mass emigration following outplantation (Steele, 1996). Similarly, consistency in group sizes throughout the growth phase indicates that fish did not migrate from colonies. Predation is considered the main source of post‐settlement mortality in *Dascyllus* spp. at Moorea (Holbrook & Schmitt, 2002) and in young reef fishes in general (Hixon & Carr, 1997; Steele, 1996; Steele & Forrester, 2002). Estimated mortality rates (*ca.* 52.4% in three days) of predator‐exposed groups were higher than previous estimates for newly settled *D. aruanus*at Moorea (*ca.* 50% in two weeks: Schmitt & Holbrook1999b, b) likely due to our focus on an earlier, higher‐mortality part of the post‐settlement period (Doherty et al., 2004; McCormick & Hoey, 2004; Steele & Forrester, 2002).

Differences in size and condition of *D. aruanus* following predator exposure treatments provide evidence for directional selection on predator‐exposed groups. It was not possible to measure initial sizes of experimental fish due to their fragility at this stage. Nonetheless, our random allocation of fish to groups means that processes occurring during the mortality phase must have been responsible for the resulting differences in length and condition observed at the end. Removal of larger, thinner fish by predators provides one possible mechanism to explain these differences. Selective mortality has previously been demonstrated in recently settled *Dascyllus*spp. at Moorea (Holbrook & Schmitt, 2002; Schmitt & Holbrook1999b, b), and this model system enabled us to generate rapid mortality, minimizing the time for confounding influences of growth to accumulate. However, the possibility remains that treatment‐ or density‐related plasticity in growth during the mortality phase was responsible for the size and condition differences we observed (Peterson & Black, 1994; Steele, 1996).

Predator exposure influenced the genetic composition of cohorts, reducing heterozygosity, reducing allelic richness, increasing relatedness, and causing a small but significant shift in allele frequencies. Although microsatellites are often considered as neutral markers, selection can influence allele composition both directly, by acting on phenotypic traits controlled by the number of tandem repeats in regulatory or protein coding regions, and indirectly, through genetic linkage (Kashi & King, 2006). One function of microsatellites may even be to generate and modulate variation in quantitative traits for rapid evolutionary adaptation (Kashi & King, 2006; King, 1994; Trifonov, 1989). While it is surprising that a short period of mortality caused genetic shifts that were detectable at a relatively small number of loci, this finding is by no means unique. Similar approaches have yielded comparable results in a range of fishes (Jones & Barber, 2005; Pini et al., 2011; Planes & Romans, 2004; Vigliola et al., 2007), mollusks (LeBlanc, Tremblay, Davidson, Landry, & McNiven, 2008), and plants (Prittinen et al., 2006). The fact that shifts were detected in a short, three‐day window suggests that mortality over the entire early life period has the potential to exert substantial leverage on the composition of older life stages.

The changes in allele frequencies we observed are consistent with directional genetic shifts in loci that are directly or indirectly linked to phenotypic traits under selection. The large reduction in heterozygosity at Da494 suggests that this locus lies in a genomic region that experiences directional selection after settlement. Previous studies have generally found increases in heterozygosity through early life, attributed to superior viability of heterozygotes or “overdominance” (LeBlanc et al., 2008; Pini et al., 2011). Examples of decreases, like those observed in the current study, also exist (Planes & Romans, 2004). Our observation that relatedness increased after the mortality period can be explained by the action of predators selectively consuming fishes with similar genotypes. This contrasts with previous studies where reductions in relatedness after settlement were attributed to dissolution of family structure (Buston, Fauvelot, Wong, & Planes, 2009). The distinguishing feature of our study is that mixing among experimental groups was prevented, leaving predation mortality as the strongest modifier of group composition.

Assuming that the mortality phase did impose selection for size and condition, there are several possible reasons why subsequent growth performance was not affected. First, size is determined not only by growth rate but also by age and size at hatching. As a result, selection for size does not necessarily impose selection for growth rate, despite the strong association between these characters (Sogard, 1997; Takasuka et al., 2007). Second, even if differences in larval growth rate did produce the size variation upon which selective mortality operated, growth phenotypes may not be fixed over time if, for example, they are environmentally determined. Inter‐individual differences in growth rates driven by variation in larval growth environments would not necessarily be maintained under common conditions. Finally, even if variation in settler size resulted from differences in intrinsic growth potential, the extent and nature of these differences may depend on environmental context (Reid, Armstrong, & Metcalfe, 2011): Conditions of food, competition, and predation risk in the growth phase could mask underlying differences that are apparent in other environments or life stages. Understanding the consequences of a selection event for subsequent performance requires knowledge of the mechanisms generating variation in the phenotype under selection.

While there is a rich literature on selective mortality in early life, few studies have examined consequences for subsequent performance. We are aware of only one other direct test: In this case, a short initial period of selective mortality (artificially imposed air exposure and elevated temperature) influenced the metabolic performance and survival of *Mytilus edulis* populations over the following 10 months (LeBlanc et al., 2008). Other studies provide indirect evidence. For example, mortality of the coastal marine fish *Diplodus sargus* on rocky structures in the Mediterranean Sea involved selective removal of slow‐growing genotypes (Planes & Romans, 2004). Contrary to our current findings, these studies demonstrate the potential importance of carryover effects from early‐life mortality in mediating the performance of the surviving cohort.

Although we found no evidence to support our hypothesis that directional selection during post‐settlement mortality influences subsequent physiological performance, the role of mortality bottlenecks in determining the phenotypic composition of wild populations warrants continued investigation. In high fecundity species, high mortality at the larval–juvenile transition has great potential to influence which individuals form the adult population. Selective mortality at this stage can be high and variable, operating directly or indirectly on growth performance and a wide range of other characters. Delayed influences of this selection on performance in later life could have hidden, yet important, implications for wild populations.

## AUTHORS' CONTRIBUTIONS

B.J.C. and S.P. conceived the ideas and designed the experiments; B.J.C. collected and analyzed the data; B.J.C. led the writing of the manuscript. Both authors contributed critically to the drafts and gave final approval for publication.

## Data Availability

Raw data and code are available from the Dryad Digital Repository: https://doi.org/10.5061/dryad.np4vh0g.
